# Epigallocatechin-3-gallate alleviates type 2 diabetes mellitus via β-cell function improvement and insulin resistance reduction

**DOI:** 10.22038/IJBMS.2022.58591.13016

**Published:** 2022-04

**Authors:** Tiantian Zhu, Minghui Li, Moli Zhu, Xu Liu, Keke Huang, Wenru Li, Shuang-Xi Wang, Yaling Yin, Peng Li

**Affiliations:** 1 College of Pharmacy, Xinxiang Medical University, Xinxiang, China; 2 Henan International Joint Laboratory of Cardiovascular Remodeling and Drug Intervention, Xinxiang, China; 3 Xinxiang Key Laboratory of Vascular Remodeling Intervention and Molecular Targeted Therapy Drug Development, Xinxiang, China; 4 School of Basic Medical Sciences, Xinxiang Medical University, Xinxiang, China; # These authors contributed equally to this work

**Keywords:** Epigallocatechin-3-gallate, Insulin resistance, Insulin secretion, Pancreatic duodenal - homeobox protein-1, Type 2 diabetes mellitus, β-cell

## Abstract

**Objective(s):**

Epigallocatechin-3-gallate (EGCG) has a good therapeutic effect on type 2 diabetes mellitus (T2DM). This work was designed to explore EGCG’s effectiveness in insulin resistance (IR) and pancreas islet β-cell function in a rat model of T2DM.

**Materials and Methods:**

Eight-week-old male Sprague Dawley rats were randomly divided into 6 groups, including the Control (normal diet), Diabetes (high-sucrose high-fat [HSHF] diet combined with tail vein injection of streptozotocin [STZ] for T2DM induction) and Treatment Diabetic rats which were treated with metformin [500 mg/kg/d] or EGCG [25, 50 or 100 mg/kg/d] intragastric administration for 10 weeks. With the exception of control animals, the other groups were fed the HSHF diet. EGCG’s effects on IR and insulin secretion were assessed by measuring body weights, and fasting blood glucose (FBG), postprandial blood glucose (PBG) and insulin levels. The morphological and molecular changes of pancreas islet β-cells were examined by hematoxylin-eosin (H&E) staining, transmission electron microscopy (TEM) and immunofluorescence.

**Results:**

Rats fed the HSHF diet combined with STZ treatment had increased body weights and blood glucose amounts, accompanied by IR and impaired β-cell function, induced T2DM, and EGCG dose-dependently restored the above indicators. Additionally, EGCG upregulated the pancreatic transcription factors pancreatic duodenal homeobox protein-1 (PDX-1) and musculoaponeurotic fibrosarcoma oncogene homolog A (MafA).

**Conclusion:**

These results suggest that EGCG reduces blood glucose amounts, and improve IR and islet β-cell disorder in T2DM.

## Introduction

Type 2 diabetes mellitus (T2DM) represents an important multi-system disorder with high heterogeneity and complexity featuring chronic hyperglycemia, impaired insulin secretion and insulin resistance (IR), which causes a large number of complications and significantly affects the patient’s quality of life (1, 2). T2DM constitutes a major public health concern around the world, making up 90%-95% of all people with diabetes mellitus. By 2040, diabetics might reach 642 million (3). T2DM prevalence is substantially rising with rapid urbanization and improvements in living standards. According to current statistics, the treatment of diabetes accounts for about 12% of global healthcare costs, i.e., approximately $673 billion. There is currently no ideal treatment for type 2 diabetes or its chronic and serious complications (4), Therefore, identifying better drugs to control type 2 diabetes is crucial.

It is well-documented that chronic IR and reduced β-cell amounts and function are the main and root reason for T2DM. In the early stage of T2DM, the body’s β-cells produce large insulin quantities to maintain blood glucose stability, which leads to hyperinsulinemia. Chronic IR progresses to T2DM when β-cells cannot secrete appropriate insulin levels for compensating for reduced insulin sensitivity, which mostly results from impaired insulin secretory function and substantial β-cell loss (5). Thus, it is important to control blood glucose and repair the insulin signaling pathway. 

As early as thousands of years ago, Chinese were cognizant of tea’s health promoting effects in humans (6). Tea leaves contain polyphenolic catechins, with >10 catechins found in distinct processed teas, with epigallocatechin-3-gallate (EGCG) showing the highest abundance and making up >40% of all catechins in freshly collected tea leaves (7). EGCG attracts growing attention for its potential health benefits such as anti-inflammatory, antiviral, cardioprotective and anti-diabetes properties (8). EGCG attenuates reactive oxygen species (ROS) production induced by dexamethasone and tumor necrosis factor-alpha (TNF-α) and increases glucose uptake. EGCG improves IR in adipocytes by ROS scavenging (9). EGCG stimulates pancreatic beta-cells for postprandial insulin elevation, which improves pancreas function. In addition, cell culture assays indicated EGCG exerts its effects through suppression of adipocyte proliferation and differentiation as well as enhancement of glucose reception by the cells via protein kinase by activating Adenosine 5’-monophosphate (AMP) (10). Therefore, EGCG might be potentially utilized for diabetes treatment.

Insulin, a peptide hormone that maintains blood glucose homeostasis, is secreted only by islet β cells (11). Β-cell development and maturation are regulated by major transcription factors, including pancreatic duodenal homeobox protein-1 (PDX-1), neuron 3 and musculoaponeurotic fibrosarcoma oncogene homolog A (MafA) (12). PDX1, i.e., human insulin promoter factor 1 (IPF1), interacts with GG2, A1 and A3, synergistically inducing insulin gene upregulation with MafA (13). PDX1 represents a key transcription factor controlling islet β-cell development and function. Low PDX1 amounts mediate β-cell loss and dysfunction in diabetics (14). MafA is a member of the large Maf transcription factor family, which also includes MafB, Maf and neural retina leucine zipper. MafA and MafB are both produced by pancreatic β-cells, although only MafA drives murine β-cell function postnatally (15). MafA-deficient mice show glucose intolerance and develop diabetes. In MafA-/- mice, insulin 1, insulin 2, PDX-1, neurogenic differentiation (NeuroD) and glucose transporter 2 (GLUT2) amounts are reduced, and glucose, arginine and KCl-induced insulin secretion are remarkably altered (16) Thus, PDX-1 and MafA may be important factors in diabetes therapy.

The current work assessed EGCG’s ability to ameliorate IR and pancreas islet β-cell disturbances in rats with T2DM. Additionally, we attempted to dissect the mechanisms by which EGCG mitigated β-cell disturbances, and found that EGCG inhibited experimental diabetes triggered by a high-sucrose-high-fat (HSHF) diet and streptozotocin (STZ) administration by increasing upregulating pancreatic PDX-1 and MafA.

## Materials and Methods

***Rats***

Male Sprague Dawley rats (150~180g) were provided by the Laboratory Animal Center, Zhengzhou University, China. Animal experiments followed the National Institutes of Health Guide for the Care and Use of Laboratory Animals (National Institutes of Health, 2011), and had approval from the Xinxiang Medical University Animal Care and Use Committee.

***Animal study***

Rat housing was carried out at 25°C under a 12-hr/12-hr light-dark cycle. Adaptative housing was performed for 7 days, and rats weighing 180±15 g were randomized into 6 groups (n=6-8/group): 

All animals received the HSFH diet (sucrose/lard/rodent chow at 20:20:60) with the exception of the control group (Table 1). 

***Biochemical assays***

Animal body weights were obtained throughout the study. Ten weeks after the drug was administered, cervical dislocation was performed for euthanasia, and pancreas tissue specimens were sampled. Retro-orbital blood collection was performed, and blood specimens underwent centrifugation at 4000 rpm (15 min at 4°C) for serum isolation. Both serum and pancreas specimens were kept at 80°C for subsequent biochemical estimations.

***Estimation of fasting blood glucose (FBG), postprandial blood glucose (PBG), fasting serum insulin (FSI) and homeostasis model assessment of insulin resistance (HOMA-IR)***

FBG and PBG amounts were assessed weekly with a blood glucometer (One Touch Ultra). FSI amounts were assessed with a rat insulin enzyme-linked immunosorbent assay (ELISA) kit (Thermo Fisher Scientific, USA), as directed by the manufacturer. HOMA-IR, the gold standard for insulin resistance evaluation was obtained as HOMA-IR = [FBG (m mol/l) × FSI (mU/l)]/22.5 (20). 

***Histology***

Pancreas specimens underwent 4% formalin fixation at ambient overnight, dehydration, parafilm embedding and sectioning at 5 µm. The sections underwent routine staining with hematoxylin-eosin (H&E) for assessing the morphology and amounts of pancreas islet β-cells.

***Insulin secretion granules***

The insulin secretion granules of islet β-cells in the rat pancreas were examined with a transmission electron microscope (TEM). Pancreas samples underwent rinsing with 0.1 M phosphate buffer saline (PBS; pH=7.2). Pancreas pieces approximating 1 mm underwent fixation with 3% chilled glutaraldehyde in 0.1 M PBS at 4°C for 12 hr. This was followed by successive staining with 2% uranyl acetate and 0.2% lead acetate, before examination by TEM (it was a transmission electron microscope). 

***Immunofluorescence***

To detect molecular changes in islet β-cells, pancreas samples underwent incubation with primary antibodies targeting the transcription factors PDX-1 and MafA. Then, goat anti-rabbit DyLight 488-linked IgG H&L (DyLight® 488) were added for 1 hr at ambient. A fluorescence microscope was utilized for analysis.

***Statistical analysis***

Data (mean±SD) were analyzed with SPSS 18. Unpaired Student’s t-test and one-way ANOVA with post hoc Newman-Student-Keuls test were performed for group pair and multiple group comparisons, respectively. P<0.05 indicated statistical significance.

## Results

***EGCG administration alters the body weight in experimental rats***

**Figure 1:**
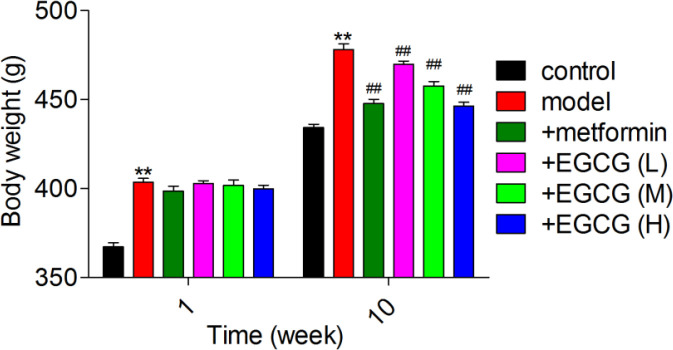
Effect of EGCG administration on rat body weights. EGCG (L), EGCG (M) and EGCG (H) stand for epigallocatechin-3-gallate at 25, 50 and 100 mg/kg, respectively. **P<0.01 vs. control; #*P*<0.05, ##*P*<0.01 vs. model. Data are mean±SD (n=6)

HSHF diet feeding and STZ injection induced diabetes. Body weights during the 10-week treatment are displayed in Figure 1. At the initial phase of diabetes, a remarkable increase in body weight was recorded, except for control animals. After ten weeks, body weights in the diabetes group were significantly decreased, and a further reduction was observed with metformin treatment. However, all doses of EGCG (25, 50 and 100 mg/kg) significantly increased the body weight.

***Effects of EGCG on blood glucose***

**Figure 2:**
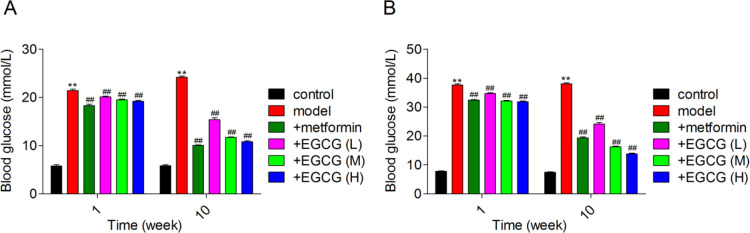
Effect of EGCG treatment on blood glucose. (A) Fasting blood glucose; (B) Postprandial blood glucose. EGCG (L), EGCG (M), EGCG (H) stand for epigallocatechin-3-gallate at 25, 50 and 100 mg/kg, respectively. **P<0.01 vs. control; ##*P*<0.01 vs. model. Data are mean±SD (n=6)

Increased FBG and PBG concentrations are two indicators of diabetes. Figure 2 represents FBG and PBG amounts in experimental animals, assessed at different times. After one week of metformin or EGCG treatment, FBG and PBG levels were decreased significantly in comparison with untreated diabetic rats. In addition, metformin’s hypoglycemic effect was much more obvious. However, after ten weeks treatment, EGCG (50 and 100 mg/kg) showed comparable or more obvious effects on hypoglycemic activity in rats with experimental diabetes. 

***Effects of EGCG on insulin levels and IR in experimental rats***

**Figure 3:**
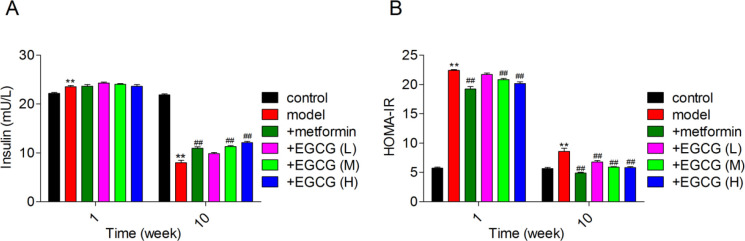
Effects of EGCG on insulin amounts and insulin resistance in diabetic rats. (A) Insulin levels; (B) HOMA-IR. EGCG (L), EGCG (M) and EGCG (H) stand for epigallocatechin-3-gallate at 25, 50 and 100 mg/kg, respectively

Figure 3 illustrates FSI amounts and HOMA-IR values in various rat groups. IR in the model group was reflected by elevated FSI and HOMA-IR in comparison with control rats. Following one week of treatment, both metformin and EGCG did not affect FSI. Meanwhile, HOMA-IR values were remarkably decreased upon administration of metformin or EGCG (50 and 100 mg/kg). With diabetes progression, FSI showed a marked reduction in the model group, while metformin and EGCG increased FSI levels. Although FSI amounts were decreased in the diabetes group, the HOMA-IR values remained high, and metformin or EGCG (both doses) caused an obvious decline in HOMA-IR values.

***EGCG restores pancreatic islet β-cell amounts and function***

**Figure 4:**
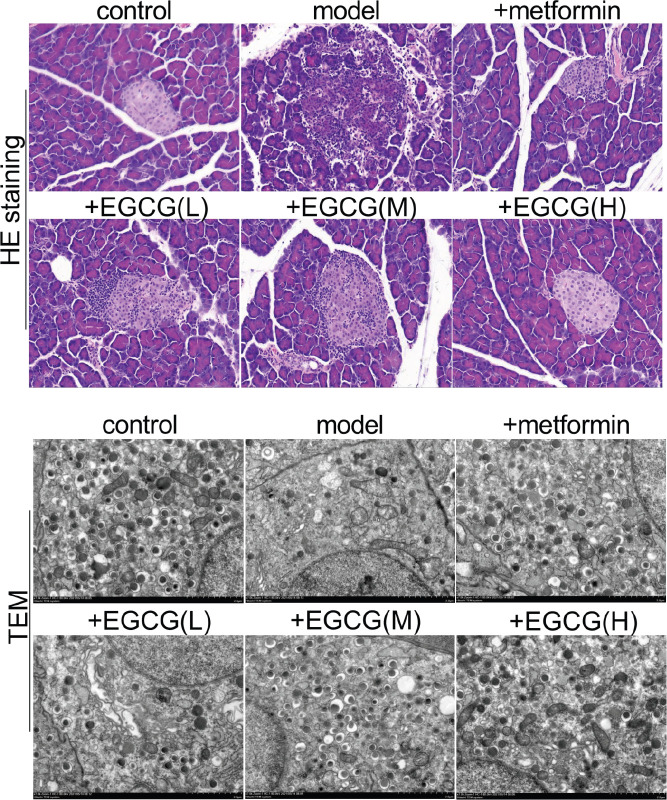
EGCG restores pancreatic islet β-cell amounts and function. (A) H&E staining of pancreas specimens (400×); (B) Insulin secretion granules in islet β-cells assessed by TEM (1000×). EGCG (L), EGCG (M) and EGCG (H) stand for epigallocatechin-3-gallate at 25, 50 and 100 mg/kg, respectively

β-cells are among multiple hormone-producing cells controlling glucose homeostasis in the islet of Langerhans. However, hyperglycemia precisely results from β-cell loss or inability to produce and secrete insulin. To assess whether the hypoglycemic effect of metformin or EGCG was due to recovered pancreatic islet β-cell amounts and function, we detected the morphology of the islet of Langerhans as well as the insulin secretion granules of islet β-cells in the rat pancreas. H&E staining showed overtly decreased amounts of pancreatic β-cells, with sparse distribution in the diabetes group. However, the number and density of β-cells were increased upon metformin and EGCG (50 and 100 mg/kg) administration (Figure 4).

***Effects of EGCG on pancreatic PDX-1 and MafA levels in experimental rats***

**Figure 5:**
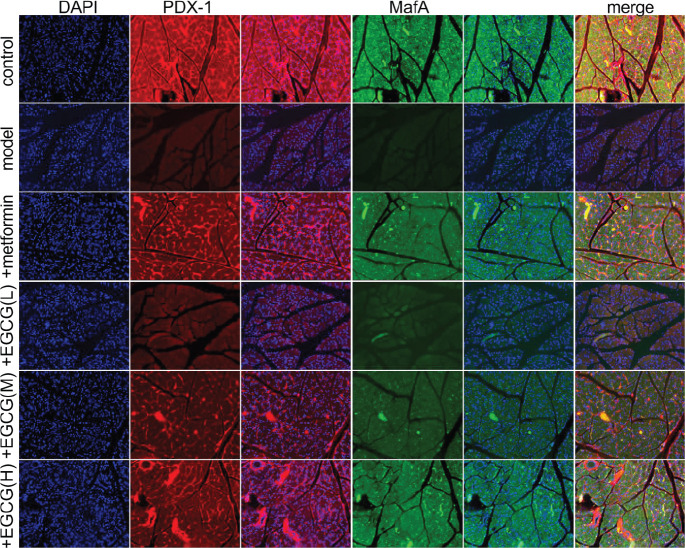
Effects of EGCG on pancreatic PDX-1 and MafA amounts in experimental rats. (A) Immunofluorescence of PDX-1 in the pancreas of rats (200×); (B) Immunofluorescent staining of MafA in the pancreas of rats (200×). EGCG (L), EGCG (M) and EGCG (H) stand for epigallocatechin-3-gallate at 25, 50 and 100 mg/kg, respectively

EGCG: Epigallocatechin-3-gallate; PDX-1: Pancreatic duodenal homeobox protein-1; MafA: Musculoaponeurotic fibrosarcoma oncogene homolog A

PDX-1 and MafA are important in the pancreas, maintaining mature β-cell function. PDX-1 or MafA silencing resulted in deteriorated β-cell function, suggesting the dynamic regulation of these transcription factors represented a cause rather than a consequence of β-cell function. Here, PDX-1 and MafA were markedly downregulated following diabetes establishment in rats. However, metformin and EGCG treatment had equivalent effects in improving the expression of these genes (Figure 5).

**Table 1:** The treatment of animal experiment

Group	Treatment
control	Animals fed rodent chow.
model	Animals fed a HSHF diet for 28 days, submitted to a 20-h fasting and administered STZ (Sigma, USA) at 30 mg/kg by tail vein injection[][]. Finally, the animals were fed the above HSHF diet for another 28 days for diabetes induction[].
+metformin	Diabetic rats were intragastrically administered 500 mg/kg metformin (dissolved in sterile distilled water as suspension) for the final ten weeks.
+EGCG (Low -dose, L)	Diabetic rats were intragastrically administered 25 mg/kg EGCG (dissolved in sterile distilled water) for the final ten weeks.
+EGCG (Medium-dose，M)	Diabetic rats were intragastrically administered 50 mg/kg EGCG for the final ten weeks.
+EGCG (High- dose，H)	Diabetic rats were intragastrically administered 100 mg/kg EGCG for the final ten weeks.

EGCG: Epigallocatechin-3-gallate

EGCG: Epigallocatechin-3-gallate

**P<0.01 vs. control; ##*P*<0.01 vs. model. Data are mean±SD (n=6)

EGCG: Epigallocatechin-3-gallate; HOMA-IR: Homeostasis model assessment of insulin resistance

H&E: Hematoxylin-eosin; TEM: Transmission electron microscope; EGCG: Epigallocatechin-3-gallate

## Discussion

As the catechin with highest abundance in green tea, EGCG has many beneficial effects compared with other known catechins, especially anti-lipid peroxidation (21), which is highly associated with neurodegenerative diseases (22), cancer (23), cardiovascular disease (24) and diabetes (25). EGCG, the most abundant bioactive component of burdock root, effectively prevents diabetes. EGCG can increase the amounts of firmicutes, Christensenellaceae and lactobacillus and decrease those of Enterobacteriaceae, which can significantly improve blood glucose homeostasis in obese diabetic mice (26). It also improves IR through GLUT2/proliferator-activated receptor γ coactivator (PGC)-1β/sterol regulatory element-binding-1c (SREBP-1c)/fatty acid synthase (FAS) signaling, which in turn enhances insulin sensitivity by improving IR-induced inflammation, oxidative damage and free fatty acid (FFA) production (27). However, the mechanism underlying the anti-diabetes effects of EGCG in HSHF diet fed and STZ injected animals has not been clearly defined. The current work showed EGCG alleviated T2DM by improving β-cell dysfunction and decreasing IR in the rat model. 

It is well known that patients with T2DM lose weight in the late stage. In our model of diabetic rats, the weights of diabetic rats were significantly elevated in the first week and declined at ten weeks, which was consistent with previous studies. However, after ten weeks of treatment with low-, medium- and high-dose EGCG (25, 50 and 100 mg/kg, respectively), animal weights were starkly elevated increased in comparison with model animals, while those of the metformin group were significantly decreased. Atypical antipsychotic drugs may, depending on the length of treatment, reduce insulin sensitivity and increase weight in healthy volunteers (28). The reason for the late-stage weight increase in diabetic rats is that treatment with EGCG may improve IR in rats (29). 

IR and metabolic syndrome constitute major risk factors for cardiovascular disease and T2DM (30). The incapacity of cells to show a response to physiological amounts of insulin is called IR, which is an etiology of T2DM (31). Recently, it was reported that IR is the main manifestation of T2DM; HOMA-IR is a recognized and validated method that uses fasting glucose amounts in blood and insulin of patients to evaluate IR (32). EGCG administration remarkably reduced blood glucose amounts and elevated body weights in diabetes-induced rats (33). Chronic administration of EGCG decreases plasma glucose amounts and alleviates insulin sensitivity in a mouse model of diabetes (34). In this research, FBG, PBG, FSI and HOMA-IR were significantly increased in diabetic animals. However, following EGCG or metformin administration, FBG, PBG, FSI and HOMA-IR were significantly decreased. Moreover, with increasing dose of EGCG, the treatment effect was significantly improved, surpassing that of metformin.

Hyperglycemia usually results from a progressive loss of insulin secretion by β-cells in the context of IR (35). IR increases blood glucose amounts, increasing the need for β-cells to synthesize and secrete higher amounts of insulin. The compensatory effect of β-cells could restore normal blood glucose in pre-diabetes at first; however, prolonged exposure to large glucose and lipid amounts eventually impairs β-cell function and kills cells, resulting in obvious diabetes (36). In the above experiments, the number of pancreatic β-cells was obviously reduced and their distribution was sparse in the diabetes group, which was consistent with previous findings. However, the number and density of these cells were increased following metformin or EGCG (50 and 100 mg/kg) administration, suggesting that EGCG and metformin treatments for repairing islet β-cells have similar effects.

It is known PDX-1 and MafA have major functions in maintaining the maturation of cells in the pancreatic duct of rats (37). Interestingly, MafA in pancreatic β-cells regulates cell specification, maturation and possibly survival/proliferation (38). PDX-1 is mainly produced by β-cells and induces β-cell differentiation and insulin mRNA production (39). It can activate tryptophan associated proteins (TRPC) 3 and TRPC6 calcium channels, induce extracellular regulated protein kinases (ERK)1/2 and cyclin D, and mediate β-cell proliferation (40). PDX-1 initially promotes the formation of the pancreas and then regulates the physiology of islet cells after maturation (41). Meanwhile, MafA expression starts at E13.5, and this protein is only found in β-cells through adulthood (42). In the pancreas of diabetic model animals, we found PDX-1 and MafA amounts were markedly reduced, while after administration of metformin or EGCG (25, 50 and 100 mg/kg, respectively), PDX-1 and MafA amounts were significantly up-regulated, suggesting EGCG and metformin have similar effects both in the early stage of pancreatic development and the main stage of insulin production.

## Conclusion

The current work confirmed EGCG alleviates T2DM by ameliorating β-cell function and decreasing IR in rats with experimental diabetes. These findings provide insights for exploring the specific mechanism of EGCG’s sugar lowering effect as well as a supportive evidence for EGCG use in clinic for T2DM treatment. 

## Authors’ Contributions

LP and YYL Study conception and design; ZTT and LMH Data analyzing and draft manuscript preparation; ZML and WSX Critical revision of the paper; LX, YX and LWR Supervision of the research; ZTT, LMH, ZML, LX, YX, LWR, WSX, YYL and LP. Final approval of the version to be published (the names of all authors must be listed). 

## Conflicts of Interest

The authors have no conflict of interest.
